# Pilot comparative effectiveness study of surface perturbation treadmill training to prevent falls in older adults

**DOI:** 10.1186/1471-2318-13-49

**Published:** 2013-05-16

**Authors:** Jon D Lurie, Alexandra B Zagaria, Dawna M Pidgeon, Judith L Forman, Kevin F Spratt

**Affiliations:** 1 The Dartmouth Institute for Health Policy and Clinical Practice, 35 Centerra Parkway, Lebanon, NH 03766, USA; 2 Dartmouth-Hitchcock Medical Center, One Medical Center Drive, 35 Centerra Parkway, Lebanon, NH 03766, USA; 3 Geisel School of Medicine at Dartmouth, Hanover, NH 03755, USA

**Keywords:** ActiveStep®, Fall prevention, Dynamic stability, Surface perturbation, Treadmill training

## Abstract

**Background:**

Falls are the leading cause of fatal and non-fatal injuries among older adults. Exercise programs appear to reduce fall risk, but the optimal type, frequency, and duration of exercise is unknown. External perturbations such as tripping and slipping are a major contributor to falls, and task-specific perturbation training to enhance dynamic stability has emerged as a promising approach to modifying fall risk. The purpose of this pilot study was 1) to determine the feasibility of conducting a large pragmatic randomized trial comparing a multidimensional exercise program inclusive of the surface perturbation treadmill training (SPTT) to multidimensional exercise alone (Standard PT); and 2) to assess fall outcomes between the two groups to determine whether an effect size large enough to warrant further study might be present.

**Methods:**

A randomized pilot study at two outpatient physical therapy clinics. Participants were over age 64 and referred for gait and balance training. Feasibility for a larger randomized trial was assessed based on the ability of therapists to incorporate the SPTT into their clinical practice and acceptance of study participation by eligible patients. Falls were assessed by telephone interview 3 months after enrollment.

**Results:**

Of 83 patients who were screened, 73 met inclusion criteria. SPTT was successfully adapted into clinical practice and 88% of eligible subjects were willing to be randomized, although 10% of the SPTT cohort dropped out prior to treatment. The SPTT group showed fewer subjects having any fall (19.23% vs. 33.33% Standard PT; p < 0.227) and fewer having an injurious fall (7.69% vs. 18.18%; p < 0.243). These results were not statistically significant but this pilot study was not powered for hypothesis testing.

**Conclusions:**

Physical therapy inclusive of surface perturbation treadmill training appears clinically feasible, and randomization between these two PT interventions is acceptable to the majority of patients. These results appear to merit longer-term study in an adequately powered trial.

**Trial registration:**

clinicaltrials.gov: NCT01006967

## Background

Falls are the leading cause of fatal and non-fatal injuries and represent a major cause of functional limitation and disability among older adults [1]. Each year in the United States an estimated 1/3 of older adults fall [2]. In 2005, 15,802 patients aged 65 and older died as the result of fall-related injuries [1]. The direct medical costs for fall-related injuries was estimated to be $19 billion in 2000 [3] and the burden of fall-related injuries is expected to grow such that by the year 2020, the total cost is estimated to become approximately $54.9 billion [4]. External perturbations such as tripping (an unexpected deceleration of the foot causing imbalance) and slipping (an unexpected acceleration of the foot causing imbalance) are a major contributor to falls. In one study of 780 fall-related hip fractures, it was found that as many as 47% were the result of a trip [5]. In a prospective study reporting the circumstances and consequences of falls for 96 older adults, 34% of the falls resulted from a trip [6].

Modifiable risk factors for falls include muscle weakness, gait and balance problems, poor vision, use of psychoactive medications, and reduction in home hazards [3]. Most effective fall-prevention interventions focus on exercise, either alone or as part of a multi-faceted program. Use of current CDC guidelines has been shown to reduce falls by as much as 37% in a healthy older adult population [7]. Although exercise appears to have efficacy in reducing falls, the optimal type, frequency, and duration of exercise remains unclear [3].

Recently, dynamic stability (the ability to maintain balance following a postural perturbation) has emerged as a potentially important aspect of defining and modifying fall risk [8]. Older adults can, with appropriate training, quickly adapt to a large postural perturbation by changing the biomechanics of their recovery [9-11]. Several researchers have focused on task-specific adaptive training (practicing the actual motor skill of a defined task such as avoiding a fall after loss of balance) with the goal of retraining the body’s inherent neuromuscular protection mechanisms to prevent a fall following a postural perturbation. There is evidence that this type of training reduces the risk of falling following simulated trips [9-11] and slips [12] and that this training can be retained over a period of months [10,12].

The ActiveStep® is a surface perturbation treadmill training (SPTT) device. In a study of healthy older adults aged 65–85, a four-week SPTT protocol reduced the incidence of falls in a simulated laboratory tripping protocol from 25% to less than 1%, and improved trunk kinematics were retained for at least four weeks [10]. However, no studies have yet assessed the clinical effectiveness of SPTT in reducing falls in the community.

The primary objective of this pilot study was to assess the feasibility of conducting a multi-center randomized trial comparing fall outcomes between two treatments: a standard evidence-based physical therapy (PT) intervention versus a similar intervention inclusive of SPTT. We hypothesized that: 1) SPTT could be incorporated into a multidimensional exercise program within a clinical PT practice; 2) patients would be generally accepting of randomization between the two treatment arms. We also sought to compare fall outcomes between the two treatment groups to determine whether an effect size large enough to warrant further study might be present.

## Methods

### Design

**Figure 1:**
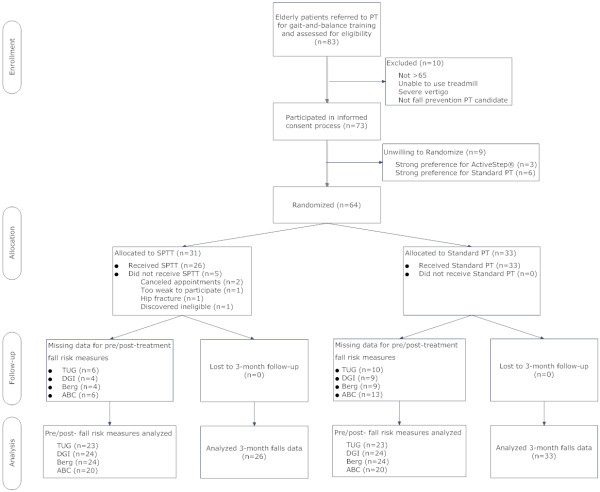
Screening, randomization, follow-up and analysis flowchart.

Figure 1 summarizes the overall study design and protocol for this randomized pilot study.

### Participants

The study was conducted in 2008 and 2009 at two outpatient PT clinics. One was a physical therapy department at a small community hospital with 2 general physical therapists; this department was an early adopter of the SPTT and had been using it clinically prior to the start of the pilot. The second site was a large academic medical center and involved 2 therapists with specific training, expertise and practice focused specifically on fall prevention, including both gait/balance training and vestibular rehab; this site had a well-established gait/balance training program but had not used the SPTT device prior to the start of the study. The Dartmouth Committee for the Protection of Human Subjects approved the study protocol.

To be eligible for the study, patients had to be 65 years or older and referred to PT because they were considered at risk to fall by their primary care provider. Eligible patients were: 1) physically able to use a treadmill; 2) willing to randomize; and 3) willing to participate in a phone interview three months after discharge from PT. Patients were excluded from the study for the following reasons: inability to use a treadmill (e.g. severe spinal issues such kyphosis, osteoporosis, or compression fractures that inhibit their ability to stand for more than a few minutes at a time); or otherwise not a candidate for gait and balance training (e.g. balance issues were purely vestibular) as determined by their physical therapist.

### Interventions

Participants were assigned using permuted block randomization stratified by site and gender to one of two treatment groups in a one-to-one ratio: 1) standard PT--an evidence-based PT program consisting of individualized exercise and mobility training based on evaluation findings; or 2) SPTT--individualized exercise and mobility training based on evaluation of findings inclusive of SPTT. Allocation concealment was ensured until after participants enrolled and completed the baseline fall risk assessment; however, the study was not blinded.

Table 1 summarizes treatment aspects of intervention in the two treatment groups. This was designed as a pragmatic, comparative effectiveness study; thus, the details of patient treatment were left to the clinical judgment of the therapist, with the exception of whether or not the SPTT was used.

**Table 1:** Summary of intervention arms

**Exercise type**	**Example**	**Treatment**	
		**Standard PT**	**SPTT (ActiveStep**®**)**
Strengthening and flexibility	• Lower extremity and trunk strengthening (*i.e.* sit to stand, heel-toe-raises, resistance bands)	X	X
	• Lower extremity stretching (*i.e.* gastrocnemius, hamstrings, hip flexors)		
Static and dynamic balance	• Challenging vestibular, visual and somatosensory system (*i.e.* altering base of support and/or surface, closing eyes, head movement)	X	X
	• Elicit ankle, hip and stepping strategies		
	• Incorporate cognitive challenge and dual task		
Mobility Training	• Assess appropriate assistive device	X	X
	• Vary gait speed		
	• Challenge surface, distance, obstacles, cognitive and dual task, stop/start		
Dynamic Stability Trip and Slip Training	• Progressively challenging anterior, posterior and lateral surface perturbation		X
	• Step response to perturbation		
	• Walking recovery after perturbation		

#### Standard PT

The treatment protocol for the Standard PT cohort consisted of an individualized multidimensional exercise program designed to address the specific impairments and functional disabilities identified during the initial assessment [13]. Since each patient presents with a different set of needs, the treatment regimen was not limited to any single form of exercise; however, each individualized program shared a number of common interventions: strengthening and flexibility exercises, static and dynamic balance exercises, and mobility training. There was no fixed time-period over which the intervention occurred; the number of sessions and the time between sessions was determined clinically by each therapist.

The interventions included both supervised in-clinic therapeutic sessions and home exercise programs. The components of the intervention prescribed for home exercise were individualized by the therapist but did not systematically differ across groups. We had no mechanism for monitoring compliance with the home exercise component of the intervention.

Strength and flexibility exercises addressed the specific deficits identified for each patient. Common exercises include gastrocnemius/soleus stretching, heel/toe raises, chair sit-to-stand, and resistive hip abduction and extension exercise. Balance exercises were designed to improve postural alignment, use of the senses for postural orientation, use of anticipatory postural adjustments, integrating sensory and motor strategies for functional balance, and to develop coordinated movement strategies. Common exercises designed to challenge somatosensory and vestibular systems included Romberg stance, modified tandem stance, tandem stance, and single limb stance. These were then advanced as tolerated by adding challenges such as head movement, opposite limb movement, and performing the tasks with eyes closed. In addition, standing balance challenges might be supplemented by the use of altered surfaces such as foam or a rocker board.

Mobility training focused on improving stability during a variety of gait tasks. Specific gait and dynamic balance exercises included walking with head turning and tilting, quick pivots, stepping over and onto obstacles, tandem walk, braiding, side steps, high steps, heel and toe walking, and fast or slow walking. Additional interventions included exercises to adjust specific gait deficits such as altering heel strike or adjusting step length. More advanced dynamic balance and gait activities may include challenge surfaces such as walking on a mat or gait tasks that are complicated by the addition of reaching or a specified cognitive task.

#### Surface perturbation treadmill training (SPTT)

Treatment for the SPTT cohort included the same general protocol as for the Standard PT cohort, but with the addition of the surface perturbation treadmill training. Each treadmill training session was incorporated into standard visits at the therapist’s discretion. The SPTT sessions consisted of the subject, who was in a safety harness that would catch them before hitting the machine if they fell, standing or walking at comfortable speed on the microprocessor-controlled, stepper motor-driven treadmill. The training protocol consisted of postural disturbances that simulated a trip (sudden backward motion of the treadmill belts that resulted in forward-directed rotation of the subject’s torso) or a slip (sudden forward motion of the treadmill belts that resulted in backward-directed rotation of the subject’s torso).

The magnitude of the disturbance for each trial is defined by four parameters: peak velocity, elapsed time to peak velocity, elapsed time during which the peak velocity is maintained, and time required to decelerate the treadmill belt to zero velocity. The magnitude of perturbation (amount and speed of sudden treadmill belt motion) was set for each trial by the therapist from level 1 (mildest) to level 5 (most vigorous) based on each patient’s day-to-day and trial-to trial performance. In general, the magnitude of the disturbances during training sessions progressed from less to more challenging; however, the exact sequence was not established *a priori*.

### Main measures

Feasibility for a larger randomized trial was assessed based on 2 major parameters: the ability to incorporate the SPTT into routine clinical practice and acceptance of study participation by eligible patients. To this end, the number of treatment sessions and session length in each group were evaluated through review of medical records. Medical records were available for 25/26 of the SPTT and 26/33 of the Standard PT subjects.

Two questions from the CDC’s Behavioral Risk Factor Surveillance System were asked to assess for falls: “The next question asks about a recent fall. By a fall, we mean when a person unintentionally comes to rest on the ground or another lower level. In the past 3 months have you fallen?” Those who reported a fall were asked a second question, “How many of these falls caused an injury? By an injury, we mean the fall caused you to limit your regular activities for at least a day or to go see a doctor” [14]. We assessed the proportion of patients in each group with any fall and the proportion with any injurious fall via phone interview at 3 months after enrollment. The early part of the assessment period thus was inclusive of the intervention, which began at the time of enrollment. Thus, differences in fall outcomes between the groups could represent differences in effectiveness for preventing falls or more rapid attainment of similar effectiveness.

Additional measures consisted of four standard instruments that assess different aspects of fall risk: 1) the Timed Up and Go (TUG) [15]; 2) the Dynamic Gait Index (DGI)[16]; 3) the Berg Balance Scale (Berg) [17]; and 4) the Activities-specific Balance Confidence scale (ABC) [18,19]. These tests were administered prior to enrollment and were repeated at time of discharge from PT.

The TUG (Timed Up and Go) is a clinician-administered test in which patients are timed (in seconds) for the following scenario: stand up from a chair, walk 3 meters, turn around, walk back and sit down. A longer time to complete the TUG indicates a lower level of functional mobility. A score of 13.5 seconds or greater indicates a fall risk in older adults [15].

The DGI (Dynamic Gait Index) assesses an individual’s ability to adapt gait to different tasks. There are eight different tasks, each scored on a four-point ordinal scale ranging from 0–3, where “0” indicates the lowest level of function and “3” the highest level of function; 24 is the highest possible score. A score of 19 or less is considered to be predictive of falls in older adults [16].

The Berg (Berg Balance Scale) consists of 14 balance items, each scored on a five point ordinal scale ranging from 0–4, where “0” indicates an inability to perform the task and “4” represents normal performance; 56 is the highest possible score. A Berg score of less than or equal to 45 is considered to be predictive of falls in older adults [17].

The ABC (Activities-specific Balance Confidence scale) measures the participant’s self-assessed confidence in being able to perform 16 specific activities without losing balance or becoming unsteady. For each task (*i.e.* walking around the house, sweeping the floor, or walking outside on an icy sidewalk) the participant indicates their level of confidence in performing the activity on a scale from 0%-100%. A higher score indicates greater confidence and higher functioning. A score of less than 67% classifies older adults as “at risk” of falling [18].

### Data analysis

We compared the demographics and baseline fall risks in the two treatment groups to evaluate the success of randomization in generating equivalent groups. The number of treatment sessions and mean session duration between treatment groups were compared using 2-sample t-tests. Chi-square tests were used to evaluate differences in falls and injurious falls between treatment groups. Given the short time frame and full follow-up of patients completing treatment, we used the proportion of subjects with any fall or any injurious fall in our analysis rather than the more complex negative binomial regression model recommended for longer-term studies with loss to follow-up [20]. Fall risk assessment scores (TUG, DGI, Berg and ABC) are continuous measures and their changes across time were evaluated within a linear mixed effects model. The Relative Risk of falls between the two groups controlling for baseline fall risk assessment scores was also assessed using generalized linear mixed models (PROC GLIMMIX). All analyses were conducted using SAS® version 9.2 (SAS Institute, Cary, NC) in the Windows XP® Professional environment.

## Results

Of 83 patients who were screened, 73 met inclusion criteria of which only 9 (12%) were unwilling to randomize due to a strong treatment preference—3 preferred treatment on the ActiveStep® and 6 preferred Standard PT. Of the 64 patients randomized, 33 were allocated to the Standard PT group and 31 to the SPTT group. Five participants were withdrawn from the study: 4 for reasons related to failure to return for treatment after enrollment, and one participant was withdrawn when it was discovered on routine data review that inclusion criteria for eligibility had not been met. All 5 of the withdrawn participants were in the SPTT group. Overall, 59 of 64 (92%) randomized patients completed treatment and were assessed for occurrence of falls 3 months after the end of treatment.

Table 2 summarizes the basic demographics of the two treatment groups. Overall, the average patient age was 80.5 years (range 65 to 96), 59.3% percent of the patients were female, and all participants were Caucasian. There were no statistically significant differences in baseline characteristics between groups in this small sample.

**Table 2:** Baseline characteristics of study participants

**Characteristic**	**SPTT (n = 26)**	**Standard PT (n = 33)**	**p -value**
Age (mean in years)	81.1	79.2	0.12
SD	6.53	7.65	
Range	66-94	65-96	
Sex - n female (%)	13 (50%)	22 (67%)	0.20
Caucasian (%)	100	100	0.99

Detailed information on the interventions was available for 51 subjects. Characteristics are summarized in Table 3. The number and duration of sessions was similar between the two groups. On average, patients in the SPTT improved by one level of perturbation difficulty over the course of treatment. Table 4 shows pre- and post- treatment results for the standard fall risk measures. No significant differences existed at baseline between the SPTT group and the Standard PT group for fall risk assessment scores. Both treatment groups demonstrated significant improvement from baseline to end of treatment with no significant differential improvement across treatment groups.

**Table 3:** Intervention details

	**SPTT (N = 25)**	**Standard PT (N = 26)**	**p-value**
**Number of PT Sessions**			
**Mean**	5.84	7.38	0.20
**Range**	1 - 19	3 - 17	
**Average Session Duration (min)**			
**Mean**	44.25	42.75	0.17
Mean Baseline Trip Level*	2.44	NA	
**Mean Final Trip Level**	3.44	NA	

* In the Surface-Perturbation Treadmill Training group, Trip level ranging from 1 (mildest) to 5 (most vigorous) refers to the magnitude of the perturbation successfully overcome without a step during that training session.

**Table 4:** Pre- and post- treatment fall risk assessment measures

		**Treatment**								
		**SPTT (N = 26)**				**Standard PT (N = 33)**				
Variable		Pre-	Post-	Diff_1_	p*	Pre-	Post-	Diff_2_	p*	p^†^
TUG	mean	14.64	11.26	−3.38	.001	14.29	11.69	−2.60	.001	.464
	(SD)	(8.69) N = 20	(6.63)	(3.56)		(4.40) N = 23	(2.87)	(3.44)		
DGI	mean	16.73	20.77	4.05	.001	17.29	20.90	3.60	.001	.657
	(SD)	(8.69)	(2.83)	(4.13)		(2.85)	(2.56)	(2.40)		
		N = 22				N = 24				
Berg	mean	45.91	50.82	4.91	.024	43.54	48.00	4.46	.032	.878
	(SD)	(6.84)	(4.63)	(4.65)		(5.90)	(11.21)	(12.82)		
		N = 22				N = 24				
ABC	mean	61.80	73.79	12.00	.001	56.55	70.00	13.45	.001	.700
	(SD)	(14.87)	(14.26)	(12.56)		(16.07)	(13.54)	(11.02)		
		N = 20				N = 20				

* Test of Null Hypothesis (H_0_: Pre- = Post-), Pre- = pre-treatment, Post- = post-treatment.

**†** Test of Null Hypothesis (H_0_: Diff_1_ = Diff_2_), Diff_1_ = SPTT (Post-) – (Pre-), Diff_2_ = Standard (Post-) – (Pre-).

TUG = Timed Up and Go Test; DGI = Dynamic Gait Index; Berg = Berg Balance Scale; and ABC = Activities Specific Balance Confidence scale; SPTT = Surface Perturbation Treadmill Training.

**Figure 2:**
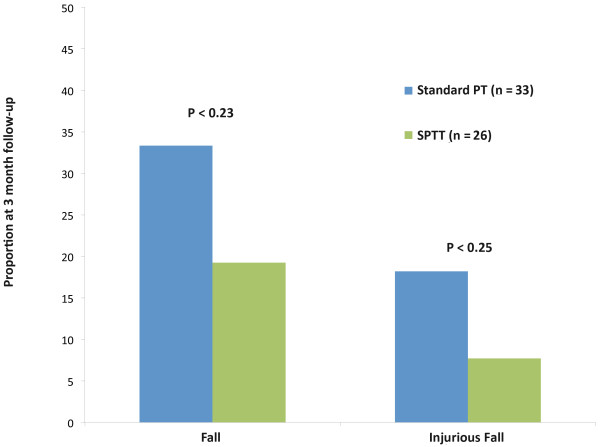
Proportion of subjects in each group with any fall and any injurious fall at 3-month follow-up.

Over a period of 3 months, 5 of 26 (19.2%) subjects in the SPTT group and 11 of 33 (33.3%) subjects in the Standard PT group had fallen at least once (Chi2 = 1.46; p < 0.23); 2 of 26 (7.7%) in the SPTT group and 6 of 33 (18.2%) in the Standard PT group had an injurious fall (Chi2 = 1.37; p < 0.25) (see Figure 2). The relative risks of falling (RR 1.61; 95% CI 0.53 – 4.88) or falling with injury (RR 1.72; 95% CI 0.27 – 11.14) was higher in the Standard PT adjusting for baseline fall risk scores, but none of these differences were statistically significant in this small pilot study.

## Discussion

This pilot comparative effectiveness study found that surface perturbation treadmill training (SPTT) could be incorporated into a multi-faceted fall prevention therapy program without an increase in the number of sessions or the average length of each session. The randomized comparison between the two PT interventions was well accepted by patients with 88% of eligible subjects willing to enroll. Standard measures of fall risk were reduced equally in the two groups, suggesting no decrement in the effect of standard PT due to substitution of some of the therapy time by SPTT. Risk of falls was reduced by a substantial amount in the SPTT group; however, these differences were not significant in this underpowered pilot study. The potential that these findings might be maintained in a larger population merits further research into the effectiveness of fall prevention therapy incorporating SPTT. The fact that the 4 patients who did not complete the study were all in the SPTT group raises the possibility that a subset of patients may find this technology intimidating which will need to be considered in planning future studies.

The findings in our study are generally consistent with the limited prior work assessing SPTT. One small study in patients with Parkinson’s disease (n = 18) demonstrated a 50% reduction in falls in the two weeks following an 8-week, 24 session treadmill-based perturbation training program [21]. This study was limited in scope but demonstrated the capability of task-specific training to reduce the actual incidence of falls in a target population. Additionally, research has demonstrated that the motor learning skills acquired on the treadmill are transferred to actual tripping situations over ground in the laboratory [9,10].

Perhaps the most intriguing finding in this study was the substantial difference in the number of patients who had a fall or injurious fall despite the similarities in the fall risk measures between groups. While this may be due to chance, given that the study was not powered to detect differences in fall rates between the two groups, the apparent discrepancy is consistent with the mechanism of the intervention. SPTT is task specific training meant to improve the response to a trip or slip and improve the chance of recovery. These tasks are not tested in any of the standard fall-risk measures such as the TUG or the BERG; thus, the finding that their specific fall risks may have improved more than their apparent overall balance and gait performance is intriguing but needs to be investigated further in an adequately powered study.

Another notable feature is that the average fall-risk scores in both treatment groups went from above the “at-risk” threshold levels at enrollment to below these levels at the end of treatment. This indicates that PT goals were met in both the intervention and control groups; this is not unexpected as the therapists involved were experienced in gait/balance training. This finding does underscore, however, that the improvements in fall outcomes being evaluated were over and above those found from a robust, standard of care intervention.

One major limitation of this study was that, as a pilot, it was not adequately powered to detect important changes in fall outcomes. A sample size calculation done for planning a larger more definitive trial showed that with a baseline fall risk in the control population of 33%, similar to what we found in this pilot, to obtain 80% power with a type one error rate of 0.05, 106 subjects would be needed to detect a 50% reduction in fall risk, 320 to detect a 30% reduction in fall risk, and 750 to detect a 20% reduction in fall risk. Thus a definitive study would need to be designed for between 500 and 1000 subjects.

A second major limitation was the reliance on patient recall at 3-month follow-up for identifying falls due to limited resources for this pilot study. While not entirely analogous to our method, 12-month recall compared to weekly post-cards to assess falls showed a sensitivity of 89% and specificity of 95%; sensitivity of 12-month recall for an injurious fall was 100% [22]. Therefore, we likely missed some falls using a 3-month recall method but there is no reason to believe that these missed falls would have been systematically biased between groups. False positive fall reports were unlikely to be a significant issue given the high specificity of recall. A more definitive study should try to improve ascertainment using diaries, more frequent monitoring, or both.

Another limitation of this study was the inability to blind testers to treatment group allocation. Since there is no adequate sham control for the SPTT, blinding of the patient or therapist was not possible. An additional limitation is the lack of standardization of the intervention dose—this was left to the discretion of the physical therapist; however, this was done by design in order to allow for individualization of the treatment based on patient capabilities and response as normally occurs in clinical practice. A final limitation worth noting is the relatively short follow-up in this pilot study: participants were followed for only 3 months. To assess the real effectiveness of SPTT, longer-term trials will be needed. If SPTT is indeed effective in fall prevention, it is clinically relevant to know how long the treatment effect might last.

## Conclusions

Preliminary findings of this pilot study show promise that a PT intervention inclusive of surface perturbation treadmill training (SPTT) may be useful within a multidimensional approach for lowering fall risk. This pilot study demonstrated the feasibility of incorporating SPTT into clinical practice and the ability to randomize a reasonable proportion of eligible patients. In addition, the effect size for reducing fall and injurious falls appears to merit consideration of longer-term studies involving larger and more diverse patient populations to assess this promising addition to balance training in older adults.

## Consent

Written informed consent was obtained from the patients for publication of this report.

## Competing interests

The ActiveStep® was developed by Simbex LLC in part with funds from NIH R44AG023407. Neither the authors nor their institution has any direct or indirect financial interest in Simbex, LLC, or received any compensation from Simbex, LLC or any other commercial interest in conducting this research. No authors have any financial or other conflicts of interest associated with this study.

## Authors’ contributions

JDNL is the principal investigator and designed and directed the study, interpreted the data, and wrote the first and subsequent drafts of the manuscript. AZ was the project manager and was instrumental in acquiring data, interpreting data, and revising the manuscript for content. DP helped design the intervention of the study, acquired clinical data and helped in its interpretation, and revised the manuscript for content. JLF was the project coordinator and helped design the study, acquire and interpret data, and revised the manuscript for content. KSF helped design the study, designed and performed the data analysis, interpreted the data, and revised the manuscript for content. All authors gave final approval of the final version.

## Pre-publication history

The pre-publication history for this paper can be accessed here:

http://www.biomedcentral.com/1471-2318/13/49/prepub
