# Correction to: Pilot comparative effectiveness study of surface perturbation treadmill training to prevent falls in older adults

**DOI:** 10.1186/s12877-022-02966-z

**Published:** 2022-05-09

**Authors:** Jon D. Lurie, Alexandra B. Zagaria, Dawna M. Pidgeon, Judith L. Forman, Kevin F. Spratt

**Affiliations:** 1 The Dartmouth Institute for Health Policy and Clinical Practice, 35 Centerra Parkway, Lebanon, NH 03766 USA; 2 Dartmouth-Hitchcock Medical Center, One Medical Center Drive, 35 Centerra Parkway, Lebanon, NH 03766 USA; 3 Geisel School of Medicine at Dartmouth, Hanover, NH 03755 USA

**Correction to: BMC Geriatr 13, 49 (2013)**

https://doi.org/10.1186/1471-2318-13-49

After publication of this article [1], the authors reported that the manuscript incorrectly identifies the trial registration for this study. It identifies the associated trial registration as NCT01006967, which was a subsequent trial that was ongoing at the time of submission, as opposed to the correct trial registration NCT00810082 which is the study described in the manuscript.

The original article [1] has been updated.
